# An Electrochemical Enzyme Biosensor for 3-Hydroxybutyrate Detection Using Screen-Printed Electrodes Modified by Reduced Graphene Oxide and Thionine

**DOI:** 10.3390/bios7040050

**Published:** 2017-11-11

**Authors:** Gonzalo Martínez-García, Elena Pérez-Julián, Lourdes Agüí, Naomí Cabré, Jorge Joven, Paloma Yáñez-Sedeño, José Manuel Pingarrón

**Affiliations:** 1 Departamento de Química Analítica, Facultad de CC. Químicas, Universidad Complutense de Madrid, E-28040 Madrid, Spain; quimigon@gmail.com (G.M.-G.); elena.epj@hotmail.com (E.P.-J.); malagui@quim.ucm.es (L.A.); pingarro@quim.ucm.es (J.M.P.); 2 Unitat de Recerca Biomèdica (URB-CRB), Antic Hospital Universitari de Sant Joan c/Sant Joan s/n, 43201 Reus, Spain; noemi.cabre@gmail.com (N.C.); jjoven@grupsagessa.com (J.J.)

**Keywords:** 3-hydroxybutyrate, graphene, thionine, electrochemical biosensor, diabetic ketoacidosis

## Abstract

A biosensor for 3-hydroxybutyrate (3-HB) involving immobilization of the enzyme 3-hydroxybutyrate dehydrogenase onto a screen-printed carbon electrode modified with reduced graphene oxide (GO) and thionine (THI) is reported here. After addition of 3-hydroxybutyrate or the sample in the presence of NAD^+^ cofactor, the generated NADH could be detected amperometrically at 0.0 V vs. Ag pseudo reference electrode. Under the optimized experimental conditions, a calibration plot for 3-HB was constructed showing a wide linear range between 0.010 and 0.400 mM 3-HB which covers the clinically relevant levels for diluted serum samples. In addition, a limit of detection of 1.0 µM, much lower than that reported using other biosensors, was achieved. The analytical usefulness of the developed biosensor was demonstrated via application to spiked serum samples.

## 1. Introduction

Blood ketone testing is considered a useful tool to detect potentially life-threatening ketoacidosis. The levels of 3-hydroxybutyrate (3-HB), one of the major ketone compounds in blood, are established between 1.1 and 3 mM in clinically significant hyperketonemia and above 3 mM in diabeticketoacidosis (DKA) [1,2]. Increased 3-HB levels in blood serum result from fatty acids degradation occurring when the body uses these acids instead of carbohydrates toobtainenergy. This situation appears when the individual is undergoing long periods of physical exercise, vomiting or fasting, as well as in type I diabetes mellitus patients with insulin deficiency. It is well known that uncontrolled diabetes can lead to the production of acetone (2%), acetoacetate (20%), and 3-HB (78%) from fatty acid catabolism [1]. Moreover,3-HB is one of the biological markers with altered values in patients with morbid obesity, the determination of this marker having a great interest in the control of this and related diseases [3].

On the other hand, it is widely accepted that the excellent properties of graphene such as large surface area, good electrical conductivity, wide potential window, low resistance to charge transfer, good electrocatalytic activity, and ease of functionalization and mass production, make this nanomaterial an excellent choice to construct electrochemical scaffolds to be used in the preparation of sensitive and reliable electrochemical biosensors. Furthermore, graphene possesses a high density of defects at the edges, which act as active points for the electron transfer to biomolecules such as redox enzymes [4]. Despite these unique characteristics, the use of graphene in the construction of enzyme biosensors is limited by the low solubility of this nanomaterial in polar and nonpolar solvents and the absence of functional groups, which makes the efficient immobilization of biomolecules difficult. Chemical modification of graphene is used to partially solve these drawbacks. For example, the presence of hydrophilic oxygenated groups in graphene oxide (GO) decreases the interaction between graphite layers, although the formation of structural defects and vacancies that interrupt the carbonaceous sp^2^ network leads to a worsening of the nanomaterial electronic properties. Reduction of GOrestores a large part of the conductivity, while some defects are not eliminated, and can minimize the problems of low reactivity [5]. The reduction of the oxygenated groups in GO can be performed electrochemically [6] or chemically using any reducing agent such as hydrazine, sodium borohydride, *p*-phenylenediamine, hydroquinone, or sodium hydrosulfite [7]. In order to avoid the use of toxic reagents, and perform a green reduction, Zhang et al. proposed the use of l-ascorbic acid as the reducing agent [8]. Although the resulting rGO still contains oxygenated functional groups, they are in much smaller number than those in the original GO suchthat this material combines both graphene and GO characteristics showing good conductivity and providing sites for anchoring to enzymes or other species specific for detection applications [9].

Few electrochemical biosensors have been reported in the literature for the determination of 3-HB. Table 1 shows thatmost of them used the enzyme 3-hydroxybutyrate dehydrogenase (3-HBDH) and involved the NAD^+^/NADH system. The enzyme 3-HBDH specifically catalyzes the conversion of the analyte to acetoacetate (AA), leading to the production of NADH, which is anelectroactive detectable product. The inherent problems related to the large overvoltage for the electrochemical oxidation of NADH, as well as the fouling of the working electrode have been minimized using different electrode materials and redox mediators. In this context, the immobilization of 3-HBDH onto screen-printed electrodes modified by coenzyme functionalized carbon nanotubes was used [10]. The electrocatalytic effect of carbon nanotubes allowed the detection of NADH to be carried out at a potential of −0.15 V vs. Ag/AgCl, and the determination of 3-HB between 10 and 100 μM witha limit of detection (LOD) value of 9 μM. An enzyme-based Clark electrode was used in a bienzyme configuration with 3-HBDH and salicylate hydroxylase (SHL). In this biosensor, once 3-HB was dehydrogenated in the presence of 3-HBDH and NAD^+^, the generated NADH was consumed by reaction with salycilate catalyzed by SHL in the presence of oxygen. Consumption of dissolved oxygen was monitored amperometrically for the determination of 3-HB [11]. A disposable amperometric biosensor with 3-HBDH immobilized on screen-printed carbon electrodes (SPCEs) was prepared using a layer of carboxymethylcellulose (CMC) hydrophilic gel to adsorb the enzyme alongside NAD^+^ cofactor and Fe(CN)_6_^3−^ as the electron transfer mediator [12]. Other redox mediators such as 1,10-phenanthroline quinone [13] or Meldola Blue [14] have also been used. Recently, an electrochemical biosensor prepared with paper electrodes and 1,10-phenantroline-5,6-dione as the redox mediator was reported for the determination of 3-HB in blood [15]. Moreover, a configuration using [Ru(bpy)_3_]^2+^ and graphene oxide was described by Veerapandian et al. for the determination of 3-HB in cow serum [16]. A screen-printed electrode fabricated with iridium–carbon particles was also used for the determination of 3-HB taking advantage of the electrocatayzed responses of NADH at the modified material [17].

In this work, we describe an electrochemical platform prepared with SPCEs modified with reduced graphene oxide (rGO) for the construction of a 3-HBDH enzyme biosensor and detection of NADH in the presence of NAD^+^, using thionine (THI) as the redox mediator. Graphene-modified electrodes have been demonstrated to provoke a remarkable enhancement in the electron transfer rate of NADH oxidation probably because of the existence of defects providing many active sites at the electrode surface [18,19]. Moreover, among the different redox mediators used to electrocatalyze the electrochemical oxidation of NADH, THI has been shown to decrease the corresponding detection potential up to −0.1 V on CNT-modified electrodes [20]. The as prepared enzyme bioelectrode allows for the determination of 3-HB at clinically relevant levels in diluted human. 

## 2. Experimental

### 2.1. Apparatus

Amperometric measurements were made with a BAS electrochemical analyzer model 100 B (1308 series) coupled to a Faraday cage and a current amplifier PreAmplifier PA-1 provided with the BAS 100/W electrochemical analysis software. Impedimetric measurements were made using a µAutolab type 3 potentiostat controlled by the FRA2 software (Ecochemie). This instrument was also used for voltammetric measurements using the GPES software (Ecochemie). Screen-printed carbon electrodes (SPCEs, 110 DRP, 4 mm Ø, were purchased from DropSens (Oviedo, Spain). These electrodes include a silver pseudoreference electrode and a carbon counter electrode. SPCEs modified with rGO and thionine were used as the working electrodes. A precision Crison Basic 20+ pH-meter, a P-Selecta Ultrasons ultrasonic bath, a P-Selecta Digaterm 100 thermostatic bath, a Vortex Wizard (VELP Scientifica, Usmate, Italy) stirrer, and a centrifuge MPW-G5R MedInstrument were also used.

### 2.2. Reagents and Solutions

The enzyme 3-hydroxybutyrate dehydrogenase (3-HBDH) from *Pseudomonas lemoignei* lyophilized powder, ≥200 units/mg protein, and thionine were from Aldrich (Madrid, Spain). 3-HB (Alfa Aesar, 98%), NAD^+^ (Gerbu, 99.6%), and NADH (Sigma, 97%) were also used. Graphene oxide (NIT.GO.M.140.10) was from (Madrid, Spain). Ascorbic acid (Gerbu, >99%) and ammonia solution (Scharlau, 32%) were used for the preparation of rGO. Sodium citrate (99%), glutamine (99%), glutamate (98%), succinate (98%), uric acid (99%), and glucose (99.5%), all from Sigma (Madrid, Spain), were tested as interferents. A 0.1 M phosphate buffer solution of pH 7.0 (PBS) was prepared from sodium di-hydrogen phosphate (Scharlau, 98%–100%) and di-sodium hydrogen phosphate (Scharlau, 99%). Before using, the buffer solutions were deoxygenated by bubbling nitrogen for 15 min. De-ionized water was obtained from a Millipore Milli-Q purification system (18.2 MΩ cm).

### 2.3. Samples

Samples analyzed were lyophilized human serum (S2257, Sigma, Madrid, Spain) spiked with 0.033 and 0.290 mM 3-HB.

### 2.4. Procedures

#### 2.4.1. Preparation of Enzyme Biosensors

A 1 mg mL^−1^ GO aqueous dispersion in de-ionized water was prepared by ultrasonic stirring for 4 h, and then centrifuged at 10,000× *g* for 10 min. The precipitate was discarded and the supernatant liquid was treated with a 25% NH_3_ solution up to pH 9–10. Further reduction of GO was performed in the presence of 2 mM ascorbic acid by maintaininga temperature of 100 °C for 15 min. Then, rGO dispersion was left in the dark at room temperature. The product was replenished every week although it was stable for at least two weeks. Finally, a 10 µL aliquot of rGO was dropped onto the electrode surface and allowed drying at room temperature. The redox mediator, thionine, was subsequently incorporated onto the rGO/SPCEs surface by deposition of 15 µL of a 1 mM THI solution and incubation for 30 min at room temperature in humid ambient. The modified electrodes were washed with de-ionized water and dried with nitrogen stream. Then, 3-HBDH/THI/rGO/SPCEs were prepared by casting 2 µL of a 100 U/mL enzyme solution onto THI/rGO/SPCEs surface and allowed drying at 4 °C. Amperometric measurements with the as prepared biosensor were carried out by depositing 50 μL of a 0.1 mM NAD⁺ solution in phosphate buffer of pH 7.0 and applying a detection potential of 0.0 V vs. Ag pseudoreference electrode. After reaching a constant current base line, 2 μL of 3-HB standard solution or the sample were added, and the steady state oxidation current was measured.

#### 2.4.2. Analysis of Human Serum

Lyophilized serum was reconstituted by dissolving 37 mg of the solid in 500 µL of deionized water. Then, the same procedure described in Section 2.4.1 was followed. In this case, 25 µL of a 0.2 mM NAD^+^ solution in phosphate buffer of pH 7.0 were deposited, and, once the background current recorded at 0.0 V was stabilized, 25 µL of spiked serum were added and the corresponding oxidation current measured. The determination of 3-HB was accomplished by applying the standard additions method, which involved four successive additions of 0.020 mM 3-HB each.

## 3. Results and Discussion

**Figure 1:**
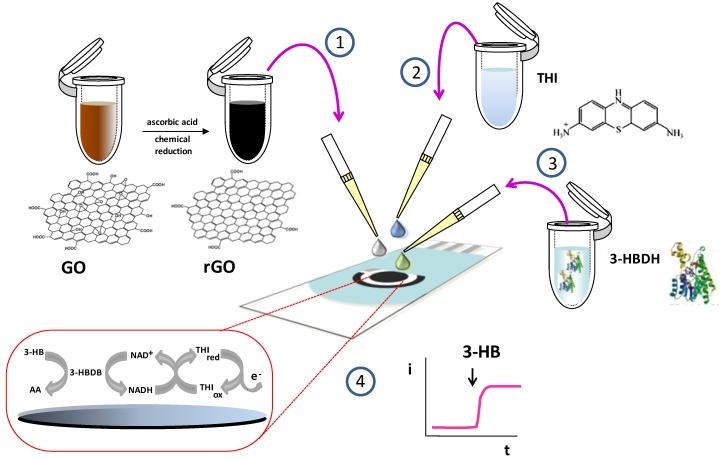
Scheme of the steps involved in the preparation and functioning of the 3-hydroxybutyrate dehydrogenase (3-HBDH)/thionine (THI)/reduced graphene oxide (rGO)/screen-printed carbon electrode (SPCE) biosensor.

Figure 1 shows schematically the steps involved in the preparation and functioning of the electrochemical enzyme biosensor. As indicated in Section 2.4.1, rGO was firstly prepared from commercial GO using ascorbic acid as the reducing agent [21]. Step 1 involved dropping a 10μL aliquot of therGO dispersion on the SPCE surface. Thereafter, the electrode was modified sequentially with 15 μL THI (Step 2) and 3-HBDH (Step 3) solutions. Amperometric responses were obtained according to the sequence of reactions shown in Figure 1 after addition of NAD^+^ and the 3-HB standard solution or the sample. Once the enzyme reaction takes place, the generated NADH chemically reduces THI and the electrochemical oxidation of THI_red_ was amperometrically monitored at 0.0 V vs. Ag pseudo reference electrode.

### 3.1. Optimization of the Variables Involved in the Preparation and Performance of the Biosensor

**Figure 2:**
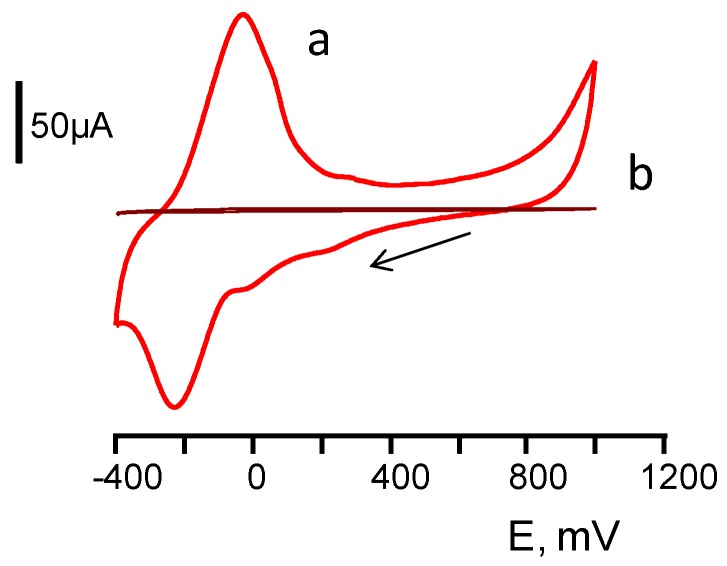
Cyclic voltammograms from 1000 to −400 mV of THI/rGO/SPCE (**a**) andrGO/SPCE (**b**) in a 0.1 mol L^−1^ phosphate buffer solution of pH 7.0. See the text for more information.

The variables involved in the preparation of the 3-HBDB/THI/rGO/SPCE biosensor were optimized. The detection potential was selected taking into account the electrochemical behavior of THI at the rGO/SPCEs. Figure 2 shows cyclic voltammograms of rGO/SPCE (Curve a) and rGO/SPCE modified by dropping 15 μL of 1 mM THI (Curve b) in a 0.1 M phosphate buffer solution of pH 7.0. As can be seen, anodic and cathodic peaks of THI appeared respectively at 0.0 V and −0.2 V corresponding to the oxidation and reduction responses of the redox mediator. Thus, 0.0 V was chosen as the optimum potential to be used.

In order to investigate the electrochemical response of THI at the electrode surface, a study of the effect of the potential scan rate in the 5–200 mV/s range was performed on the CVs of THI/rGO/SPCE. Both oxidation and reduction peak currents showed a linear dependence with the scan rate (*R*^2^ = 0.992 (ox) and *R*^2^ = 0.995 (red)), whereas curves were obtained by plotting current vs. square root of the scan rate. Moreover, the log i vs. log v plots were linear (*R*^2^ = 0.992 (ox) and *R*^2^ = 0.996 (red)) with slope values of 1.09 (ox) and 1.03 (red). These results demonstrated that the electrochemical response of THI is surface-controlled.

Regarding the rGO loading on the SPCE, the selected value was that optimized previously by our group [22]. Using these experimental variables, calibration plots of NADH at THI/rGO/SPCE in the 0.1–1.0 mM range were obtained following the equation I, μA = (0.19 ± 0.01) [NADH, mM] + (0.018 ± 0.007) (*R*^2^ = 0.990). For comparison, the value of the slope is approximately 15times greater than that found in the literature using a screen-printed electrode fabricated with iridium–carbon particles (m = 0.013 μA/mM) and a potential value of +0.2 V vs. Ag/AgCl [17].

**Figure 3:**
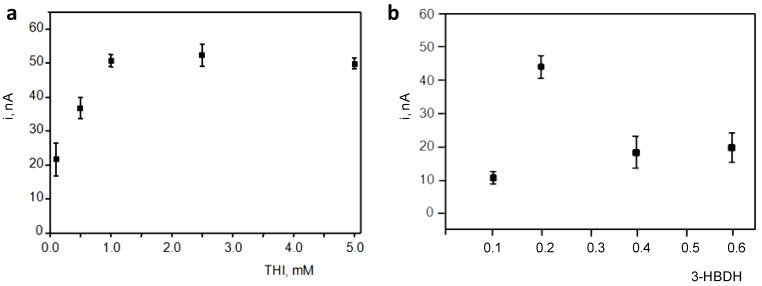
Effect of the THI and enzyme loadings on the amperometric responses measured with THI/rGO/SPCE (**a**) and 3-HBDH/THI/rGO/SPCE biosensors (**b**).

The effect of the THI loading was checked by measuring the current from a 0.2 mM NADH solution at THI/rGO/SPCEs prepared with different THI concentrations in the 0.1–5.0 mM range. As is shown in Figure 3a, a sharp increase in the oxidation current was observed when the THI concentration increased up to 1 mM and leveled off for larger concentrations. Using 1 mM THI, an incubation time of 30 min was found to be sufficient to ensure a successful adsorption of the redox mediator. The effect of the enzyme loading on the amperometric response of the biosensor was also evaluated by preparing biosensors with different enzyme loadings over the 0.01–0.6 units range in the presence of 0.1 mM NAD^+^ and 0.040 mM 3-HB. Figure 3b shows thatoptimal responses were obtained with 0.2 3-HBDH units, which is similar to thoseemployed in other reported biosensors for 3-HB. The sharp decrease in the measured current for larger 3-HBDH loadings is most likely due to the decrease in conductivity caused by high biomolecule loadings on the electrode surface. Moreover, this optimal enzyme loading was verified upon constructing calibrations plots for 3-HB over the 0–0.1 mM range with biosensors prepared with enzyme loadings in the above-mentioned range. A larger slope value for the calibration was obtained with 0.1 U 3-HBDH.

**Figure 4:**
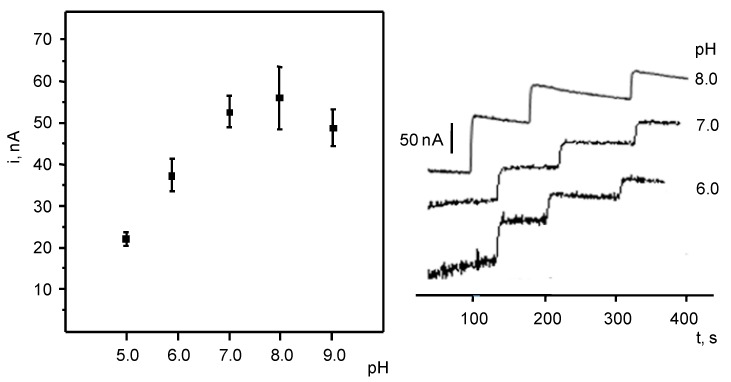
Effect of pH on the biosensor response. See the text for more information.

The effect of pH on the enzyme electrode response was also studied in 0.1M phosphate buffer solutions with pH ranging between 5.0 and 9.0. Figure 4 shows thatlarger currents were measured at 7.0–8.0 pH values. Furthermore, amperograms corresponding to additions of 20 μM NADH in the optimal experimental conditions revealed a better reproducibility of the responses recorded at pH 7.0; thus, this pH value was selected for further work. Under the optimized experimental conditions, the enzyme biosensor exhibited a rapid response reaching 95% of the maximum current in 7 s.

**Figure 5:**
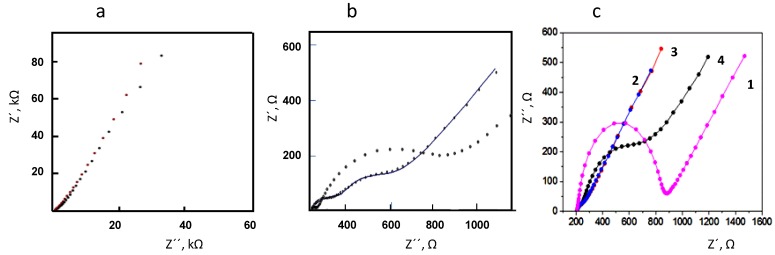
Nyquist plots recorded at (**a**) 3-HBDB/THI/rGO/SPCE (black) and 3-HBDB/rGO/SPCE (red) in 0.1 M KCl; (**b**) 3-HBDB/THI/rGO/SPCE (o oo o) and 3-HBDB/rGO/SPCE (^_____^) in 1 mM Fe(CN)_6_^3−/4−^; (**c**) SPCE (1), rGO/SPCE (2), THI/rGO/SPCE (3), and 3-HBDB/THI/rGO/SPCE (4), using 1 mM Fe(CN)_6_^3−/4−^ in 0.1 M KCl. Bias potential = 0.125 V.

Characterization of the electrode surfaces was performed by electrochemical impedance spectroscopy. As can be observed in Figure 5a, the EIS spectra obtained at 3-HBDB/rGO/SPCE and 3-HBDB/THI/rGO/SPCE biosensors in 0.1 M KCl solutions prepared in the absence of redox probe exhibited lines with the slope value much higher than 1, revealing a behavior tending to that of an ideal capacitor. No differentiated electrochemical process was observed in the presence of THI. Furthermore, when using solutions containing 1 mM Fe(CN)_6_^3−/4−^ as the redox probe (Figure 5b), both bioelectrodes showed the expected behavior for a diffusion-controlled electron transfer. Moreover, a small semicircle appeared at the higher frequencies, which is less apparent at the electrode prepared with THI. This behavior is probably due to the existence of film defects such as pinholes or to a non-uniform thickness throughout the substrate, but it cannot be attributed to a different electroactive behavior of the redox probe on the electrodes. The different steps involved in the preparation of the enzyme biosensor were also monitored by means of electrochemical impedance spectroscopy using 1 mM Fe(CN)_6_^3−/4−^ in 0.1 M KCl as the redox probe. Figure 5c shows the Nyquist plots recorded at the bare SPCE (Curve 1), rGO/SPCE (Curve 2), THI/rGO/SPCE (Curve 3), and 3-HBDB/THI/rGO/SPCE (Curve 4). As can be seen, the charge transfer resistance at the bare SPCE (638Ω) is significantly higher than that at rGO/SPCE or THI/rGO/SPCE (87 and 94 Ω, respectively) as a consequence of the presence of rGO providing a high conductivity for the modified electrode. The deposition of the redox mediator practically did not affect the electron transfer resistance. Moreover, as expected, the enzyme immobilization provoked an increase in the R_CT_ value (1547 Ω) due to the partial insulation of the electrode surface, thus confirming the successful adsorption of the protein on the modified electrode.

### 3.2. Analytical Figures of Merit of the Biosensor

**Figure 6:**
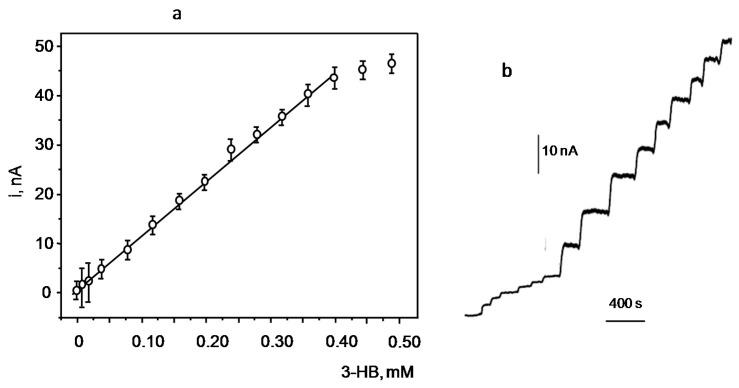
Calibration plot for 3-HB recorded with the 3-HBDH/THI/rGO/SPCE and some amperometric responses for additions of (**a**) 20 μM and (**b**) 100 μM 3-HB. Error bars are calculated according to ± s (*n* = 3).

The calibration plot for 3-HB constructed with the 3-HBDB/THI/rGO/SPCE biosensor is displayed in Figure 6. The linear range extends between 0.003 and 0.400 mM (*r*^2^ = 0.992) with a slope and intercept values of 110 ± 4 nAmM and 0.6 ± 3 nA, respectively. The LOD was calculated according to the 3s_b_/m criterion, where s_b_ was estimated as the standard deviation (*n* = 10) from the mean current of solutions containing no 3-HB, and m was the slope of the linear calibration range. An LOD value of 0.001 mM, which is significantly lower than those reported using other biosensor configurations, was obtained (see Table 1). The limit of quantification (10 s_b_/m) was 0.003 mM.

The enzyme reaction occurring at the 3-HBDB/THI/rGO/SPCE biosensor fitted well into Michaelis–Menten kinetics, as demonstrated by calculation of the “x” parameter (1.05 ± 0.07) from the corresponding Hill’s plot ([log (i_max_/1) ‒ 1] vs. the log of 3-HB concentration). Therefore, the apparent Michaelis–Menten constant (K_M_^app^) was calculated from the Lineweaver–Burk plot. The K_M_^app^ value obtained was 1.50 ± 0.03 mM. This value is significantly lower than that reported by immobilization of 3-HBDH onto a thick film screen-printed iridium-modified electrode, 2.3 mM [16], or in mesoporous silica (FSM8.0), 2.8 mM [13], and much lower than that obtained when a platinized activated carbon electrode was used as immobilization surface, 5.4 mM [23]. The low K_M_^app^ value obtained indicates a high affinity of the enzyme for the substrate when it is immobilized on the rGO-modified electrode.

Amperometric currents measured for 0.200 mM 3-HB with 10different biosensors prepared with the same protocol provided an RSD value of 4.5%, thus showing a good reproducibility both in the preparation procedure of the enzyme electrodes and in the amperometric transduction. Importantly, the analytical characteristics of the enzyme biosensor are suitable for the determination of 3-HB in human serum where concentrations are in the range of mM units [1]. When the analytical figures of merit of the enzyme electrode are compared with data provided by commercial kits for 3-HB determination, some noticeable differences become apparent. These kits claim for dynamic ranges usually covering from hundreds to tenths mM. For example, the ab83390 (colorimetric) enzymatic kit from Abcam [24] reports a 0.01–0.1 mM linear range, while the fluorimetricab180876 kit [25] provides a linear range of 0.002–0.01 mM. Therefore, it can be concluded that the range of linearity provided by the enzyme biosensor (0.003–0.400 mM) is wider. In addition, the limits of detection and quantification achieved are lower (or much lower in the case of colorimetric detection) At this point, it is important to note that the criteria used to calculate the LOD values in these kits are rarely given in the commercial protocols. Moreover, the precision levels for these kits are around 10% or higher. Summarizing, it can be concluded that the analytical performance of the developed biosensor, covering a wide linear range of 3-HB concentrations and the clinically relevant interval, improves, in general terms, the performance claimed for other approaches.

The storage stability at 4 °C ofthe 3-HBDB/THI/rGO/SPCE biosensor was evaluated by measuring in different days the amperometric responses for 0.1 mM 3-HB solutions applying the procedure described in Section 2.4.1. The results obtained (not shown) revealed that the initial response of the biosensor kept within the limits of control set at three times the standard deviation of the measurements (*n* = 10) carried out in the first working day, during at least 20 days after the biosensor preparation, thus showing a good storage stability.

**Figure 7:**
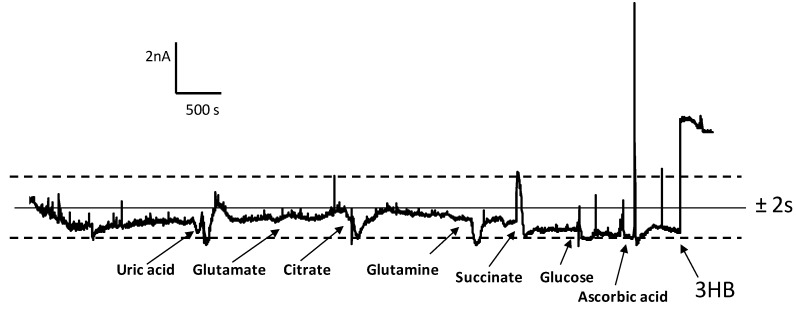
Amperometric responses obtained at the 3-HBDH/THI/rGO/SPCE biosensor for 0.178 mmol/L uric acid; 0.103 mmol/L glutamate; 0.060 mmol/L citrate; 0.067 mmol/L glutamine; 0.0083 mmol/L succinate; 0.0073 mmol/L glucose; 0.057 mmol/L ascorbic acid; 0.020 mmol/L 3-HB. Other conditions as in Figure 7.

Regarding the selectivity of the biosensor, various compounds that can be present in the 3-HB serum samples, such as citrate, glutamine, glutamate, succinate, ascorbic acid, uric acid, and glucose, were tested as potential interfering substances. The effect of their presence was evaluated by measuring the currents under the optimal experimental conditions for the 3-HBDB/THI/rGO/SPCE biosensor in the absence of 3-HB. The concentration level checked for each interfering compound was that corresponding to the normal physiological levels in serum. Figure 7 shows the amperometric responses obtained compared to that measured for a low 3-HB concentration of 0.020 mM. This figure also allows for the background noise to be visualized. As can be seen, all the potential interfering compounds except ascorbic acid, produced very slight variations in the biosensor response, all of them within the range of two times the standard deviation for the background noise. In the case of ascorbic acid, a relatively high current was observed, probably as a consequence of its oxidation at the detection potential value used. Thus, for example, after the addition of 0.057 mM ascorbic acid, a current equivalent to a 0.040 mmol/L 3-HB concentration was measured. In spite of this interference, no significant current response was apparent from human serum components when the biosensor was used for the analysis of this type of sample, thus confirming the suitability of the developed method for the enzymatic determination of 3-HB in serum.

### 3.3. Determination of 3-HB in Spiked Human Serum

The possible existence of matrix effects in human serum was evaluated by comparing the slope values of the calibration plots constructed with 3-HB standard solutions in a 0.1 M phosphate buffer, and in serum samples spiked with the analyte at concentrations ranging between 0.01 and 0.400 mM. As described in Section 2.4.2, the analytical procedure involved a 1:1 serum dilution with the 0.2 mM NAD^+^ solution in phosphate buffer of pH 7.0. Under these conditions, the slope value of the linear calibration graph obtained for spiked serum samples was 66 ± 4 nAmM, which is significantly different from the slope of the calibration plot constructed with 3-HB standards in buffered solution (110 ± 4 nA mM). Therefore, it could be concluded that a significant matrix effect provoked by the serum components occurred. However, it is important to note that no unexpected increases in the biosensor response caused by the presence of potential interfering compounds were observed. This was confirmed by the low level of the zero signal (0.4 ± 0.1 nA), which is even slightly lower than that of the calibration plot prepared with 3-HB solutions in phosphate buffer (0.6 ± 0.3 nA). Accordingly, the standard additions method wasemployed to quantify the analyte concentration is serum.

The analyzed samples were serum spiked with 3-HB at two concentration levels: 0.033 and 0.290 mM, which are lower than those expected for patients with health problems caused by hyperketonemia or diabetic ketoacidosis [1]. Other more concentrated samples could be analyzed by applying a proper sample dilution. The results obtained inquintuplicate provided 3-HB concentrations expressed as $\bar{x}\pm \left(t_{0.05}\times s\right)/\sqrt{n}$ of 0.032 ± 0.003 and 0.294 ± 0.015 mM, respectively, with mean recoveries of 98% ± 8% and 101% ± 5%. These results demonstrate fairly well the usefulness of the developed biosensor for the analysis of low 3-HB concentrations in human sera with minimal sample treatment.

## 4. Conclusions

A simple and facile method of preparing an electrochemical enzyme biosensor for the determination of the biomarker 3-HB in human serum has been constructed. The biosensor takes advantage of the well-known benefits provided by SPCEs modified with reduced graphene oxide, in terms of enhanced electron transfer rate of NADH oxidation, and the use of thionine as the redox mediator. The enzyme biosensor exhibits remarkably good analytical characteristics in terms of the range of linearity, high sensitivity, and low detection limit in comparison with theother biosensor configurations and commercial kits for 3-HB determination. Importantly, the enzyme electrode allows for the quantification of the analyte at clinically relevant levels in human sera involving minimal sample treatment (just an appropriate dilution with the working buffer solution).

## Figures and Tables

**Table 1:** Some electrochemical enzyme biosensors for the determination of 3HB.

Electrode	Biosensor Fundamentals	Technique/E Detec.	Linear Range /LOD, mM	Sample	Ref.
3-HBDB/NAD^+^/SWCNTs/SPCE	3-HB + NAD^+^ (3-HBDH)→NADH. Detect. NADH	CV/−150 mV vs. Ag/AgCl	0.01–0.1/0.009	human serum	[10]
Clark electrode	3-HB + NAD^+^ (3-HBDH)→NADH NADH+O_2_(SHL)→NAD^+^ Detect. O_2_ consumption	amperom.−600mV vs. Ag/AgCl	0.008–0.8/0.0039	spiked human serum	[11]
3-HBDB/NAD^+^/Fe(CN)_6_^4−^/CMC/SPCE	3-HB + NAD^+^ (3-HBDH)→NADH Detect. NADH with Fe(CN)_6_^4-^	amperom. +300 mV vs. Ag/AgCl	0.014–5.3/0.014	human serum	[12]
1,10-PQ/NAD^+^/3-HBDH/SPCE	3-HB + NAD^+^ (3-HBDH)→NADH Detect. NADH with 1,10-PD	+200 mV vs. Ag/AgCl	0–6/-	spiked blood	[13]
3-HBDH–FSM8.0/NAD+/MB/SPCE	3-HB + NAD^+^ (3-HBDH)→NADH Detect. NADH with MB	amperom. −50 mV vs. Ag/AgCl	0.03–8/0.0292	-	[14]
1,10-PD/NAD^+^/3-HBDH/EPAD	3-HB + NAD^+^ (3-HBDH)→NADH Detect. NADH with 1,10-PD	amperom. +200 mV	0–6/0.3	spiked whole blood	[15]
3-HBDH/[Ru(bpy)_3_]^2+^/GO/NAD^+^/SPCE	3-HB + NAD^+^ (3-HBDH) → NADH. Detect. NADH with [Ru(bpy)_3_]^2+^	amperom. +60 mV vs. Ag/AgCl	0.2–2.0/-	bovine serum	[16]
SPIrCE	3-HB + NAD^+^ (3-HBDH)→NADH Detect. NADH	amperom. +200 mV vs. Ag/AgCl (T = 37.5 °C)	0–10/-	bovine serum	[17]
3-HBDH/THI/rGO/SPCE	3-HB + NAD^+^ (3-HBDH)→NADH Detect. NADH with THI	amperom. 0 mV vs. Ag	0.003–0.4/0.001	spiked human serum	This work

CMC: carboxymethyl cellulose; EPAD: electrochemical paper-based analytical device; FSM8.0: mesoporous silica; MB: Meldola Blue; 1,10-PD: 1,10-phenanthroline-5,6-diol; 1,10-PQ: 1,10-phenanthroline quinone; SPIrC: carbon-ink containing iridium; THI: thionine; LOD: limit of detection.
